# Profiles of Nature Exposure and Outdoor Activities Associated With Occupational Well-Being Among Employees

**DOI:** 10.3389/fpsyg.2018.00754

**Published:** 2018-05-17

**Authors:** Katriina Hyvönen, Kaisa Törnroos, Kirsi Salonen, Kalevi Korpela, Taru Feldt, Ulla Kinnunen

**Affiliations:** ^1^Department of Psychology, University of Jyväskylä, Jyväskylä, Finland; ^2^Finnish Institute of Occupational Health, Helsinki, Finland; ^3^Psychology, Faculty of Social Sciences, University of Tampere, Tampere, Finland

**Keywords:** nature exposure, outdoor activities, work engagement, burnout, employees

## Abstract

This research addresses the profiles of nature exposure and outdoor activities in nature among Finnish employees (*N* = 783). The profiles were formed on the bases of nature exposure at work and the frequency and type of outdoor activities in nature engaged in during leisure time. The profiles were investigated in relation to work engagement and burnout. The latent profile analysis identified a five-class solution as the best model: *High exposure* (8%), *Versatile exposure* (22%), *Unilateral exposure* (38%), *Average exposure* (13%), and *Low exposure* (19%). An Analysis of Covariance (ANCOVA) was conducted for each well-being outcome in order to evaluate how the identified profiles related to occupational well-being. Participants with a *High, Versatile*, or *Unilateral exposure* profile reported significantly higher work engagement in the dimensions of vigor and dedication than did the participants with a *Low exposure* profile. The participants with the *High exposure* profile also reported lower burnout in the dimensions of cynicism and professional inadequacy than the participants with the *Low exposure* profile. Nature exposure during the workday and leisure time is an under researched but important aspect in promoting occupational well-being.

## Introduction

Contact with the natural environment (e.g., park walks during the workday) and nature elements (e.g., indoor plants) can have beneficial effects on general and work-related well-being, as well as on work attitudes (e.g., Brown et al., 2014; Gray and Birrell, 2014; de Bloom et al., 2017; Sianoja et al., 2017; see reviews by Korpela et al., 2015; Horr et al., 2016). Also, physical activities in natural environments during leisure time can contribute to employee well-being as was noted in a 1-year follow-up study among employees (Korpela et al., 2017). The present research builds on such previous research by incorporating nature exposure at work and including not only the frequency but also the type of outdoor activities engaged in within natural environments during leisure time. In this research, the term “natural environments” refers to green and natural areas such as parks, forests, fields, marshes, beaches, waters, playgrounds, and playing fields.

Using a person-centered approach (e.g., Wang et al., 2013; Bergman and Lundh, 2015), we aimed to identify subgroups of employees characterized by their frequency of nature exposure during working hours and leisure time and the type of outdoor activities they engage in during their visits to natural environments. We were able to identify employee profiles, categorized to be as homogenous as possible within each profile and as heterogeneous as possible between the profiles in terms of employees' nature exposure and outdoor activities in natural environments. This kind of approach is meaningful, since, in reality, people have access to various types of nature exposure and activities concurrently. We further investigated the differences between the subgroups of employees to find out which are the least and most beneficial employee profiles in terms of occupational well-being. Our research seeks to address the question of what occupational well-being benefits are associated with nature exposure and outdoor activities in natural environments, and who benefits most (Bowler et al., 2010). The results are applicable in occupational health services promoting occupational well-being and designing nature-based interventions that target occupational well-being.

### The effects of natural environments on well-being

We considered the effects of natural environments on occupational well-being within the context of the Attention Restoration Theory (ART; Kaplan and Kaplan, 1989; Kaplan, 1995, 2001) and Stress Recovery Theory (SRT; Ulrich, 1983, 1993; Ulrich et al., 1991). ART (e.g., Kaplan and Kaplan, 1989) focuses on the cognitive processes involved in information processing. Individuals use directed attention in organizing cognitive stimuli, for instance, in problem solving. According to ART (e.g., Kaplan, 2001), directed attention is a limited resource and vulnerable to fatigue. If directed attention is fatigued, the attentional restoration is suggested to be supported by certain environments that have restorative qualities. In line with ART, restoration is more likely to happen when an individual becomes fascinated and the attention is effortlessly drawn to an interesting element in the environment. Thus, the directed attention can replenish and the individual experiences attentional restoration. In addition to fascination, there are three other central elements in nature contributing to attentional restoration: having the sense of being away, the extent to which the environment allows one to engage, and compatibility between oneself and the environment.

The physiological and affective changes observed in natural environments are explained by the Stress Recovery Theory (SRT; Ulrich, 1983; Ulrich et al., 1991). Natural environments impact stress recovery on several levels that can play a key role in occupational well-being. Natural environments speed up physical recovery via releasing muscle tension and reducing blood pressure, heart rate and salivary cortisol (e.g., Kim et al., 2009; Lee et al., 2011; Tsunetsugu et al., 2013). Natural environments promote positive changes in affect and emotions (see a review by Pretty et al., 2007; Bowler et al., 2010). That is, natural environmental factors can facilitate stress recovery through autonomic nervous system changes that increase relaxation (Gladwell et al., 2012) and positive mood (e.g., Bowler et al., 2010). These theories are relevant in explaining restoration and recovery processes among employees since modern working life demands them to process extensive and complex information that burdens attention for long periods of time resulting in cognitive strain. Work environments also create psychosocial stressors (e.g., time pressure and performance expectations), resulting in the reduction of occupational well-being (e.g., Siegrist et al., 2009; Paškvan et al., 2016). Opportunities for restoration and recovery can therefore contribute to better occupational well-being among employees. In turn, when stress recovery fails, employees may experience an increase in job-related burnout.

Restoration has been shown to be more efficient in natural than in built environments (e.g., Ulrich et al., 1991; Kaplan, 1995; Herzog et al., 2002; Berman et al., 2008; Aspinall et al., 2015). A favorite place in a natural rather than in built environment can increase affect regulation, promoting positive states and stress recovery (e.g., Korpela and Ylén, 2009; Korpela et al., 2010). The restorative effects are observed when viewing or being physically active in natural environments (Elings, 2006; Stigsdotter et al., 2011; Tyrväinen et al., 2014). Natural environments, in fact, contribute to well-being beyond physical activity (Ulrich and Parsons, 1992; de Vries et al., 2003; Grahn and Stigsdotter, 2010). Research provides evidence that natural environments are not only restorative after exposure to stress and attention fatigue but also positively impact generally healthy individuals (Frumkin, 2001; Nielsen and Hansen, 2007). Natural environments can, for instance, increase physical activity- and exercise-related benefits, trigger deep reflection and strengthen the nature connection (see a review by Brymer et al., 2010). Nature exposure and outdoor activity can be used as means for the employees' psychological self-regulation toward recovery from work strain and improvement of occupational well-being and health.

### The present study

In this research, we propose that employees' level of nature exposure is related to their occupational well-being. Nature exposure during the workday was taken into account since employee well-being benefits have been observed in relation to such exposure during work (Lottrup et al., 2012; Gilchrist et al., 2015; Sianoja et al., 2017). For example, employees who took a daily 15-min park walk during their lunch break over a 2-week trial reported increased vitality, decreased fatigue and decreased blood pressure after each break in the afternoons (de Bloom et al., 2017; Torrente et al., 2017). On an individual level, on the days they took the park walk, they showed decreases in end-of-workday stress and fatigue as well as better concentration at work compared to days when they took lunch breaks without a walk through the park (Sianoja et al., 2017). In addition, nature exposure during leisure time can contribute to employees' vitality and stress recovery (Korpela and Kinnunen, 2011; Korpela et al., 2017).

We also took into consideration the types of activities that employees engaged in during leisure time, ranging from being in and enjoying nature to more physical activities of jogging or skiing. There is mixed evidence regarding the relationship between the type of outdoor activity and well-being. Some previous studies show that well-being effects of green exercise are not related to the type, intensity or length of the activity (Pretty et al., 2007). However, exceptions have been reported; for example, a longitudinal study (Korpela et al., 2017) reported that physical exercise in nature was more effective than some other, less intense activities, such as gardening. It could be that those employees who spend more time in natural environments also generally engage in more varied activities (e.g., gardening, spending time at a summer cottage, walking, skiing, picking berries) than those employees who visit natural environments only infrequently (e.g., enjoying the scenery and photography). These considerations and previous findings call for further studies and we have consequently taken into account the heterogeneity of the outdoor activities in nature.

The first research question we posed relates to whether there are distinctive profiles of nature exposure and outdoor activity in nature. Due to the exploratory nature of the person-centered analyses, we could not set firm hypotheses regarding the number of profiles or their respective levels of exposure and heterogeneity of activities. However, as we aimed to reach a large and heterogeneous sample of employees, we expected to find more than one profile such as relating to various frequencies of exposure and different activities. It is likely that the identified profiles would differ quantitatively from each other. For example, there could be a profile that reflects less frequent nature exposure at work and during leisure time as well as less varied activities in nature. It was also deemed reasonable to assume that there would be a profile that relates to more frequent nature exposure at work and during leisure time in addition to more varied activities in nature. These expectations were based on previous research that has shown that employees differ in their levels of nature exposure and participation in outdoor activities in nature (Gilchrist et al., 2015; Korpela et al., 2015). It was also thought possible that the profiles would differ from each other qualitatively, meaning that they might show different combinations of nature exposure and outdoor activities in nature. For example, while an individual's nature exposure may be high, only certain physical activities may be pronounced in his or her profile (e.g., daily walks with a dog).

The second research question focused on investigating whether the profiles would relate to occupational well-being. Previous research has indicated that exposure to a natural environment at work or during leisure time relates to employee well-being, such as vitality (Korpela et al., 2017) and mental well-being (Brown et al., 2014; Gilchrist et al., 2015). We focused on well-being at work measured by burnout and work engagement, since these two aspects of occupational well-being have not been included in previous studies in conjunction with nature exposure. These aspects measure work-related mental states, which are important in working life as modern employees are expected to work longer, extending their careers (e.g., being engaged at work but not to the point of burnout).

The psychological syndrome of burnout is typically described as exhaustion, cynicism, and reduced professional efficacy caused by prolonged job stress (e.g., Maslach et al., 1996; Maslach and Leiter, 2008). The core component of the syndrome, exhaustion, refers to the depletion of emotional and physical resources from doing one's work. Cynicism describes a negative or distant attitude toward one's work in general, and it can be characterized as dysfunctional coping through which employees detach themselves from their work. Reduced professional efficacy represents feelings of incompetence and ineffectiveness in regard to both the social and non-social aspects of occupational achievements. Work engagement, in turn, aims to capture employees' positive work-related states of vigor, dedication, and absorption at work (e.g., Schaufeli et al., 2006; Bakker and Demerouti, 2008). Vigor describes high energy and mental resilience toward work. Dedication refers to the employee's feelings of pride, meaningfulness and enthusiasm about the work. The absorption component describes being fully concentrated and immersed in work, as well as losing the sense of time while working. We also controlled for psychosocial stressors in the work environment in the form of employee efforts and rewards (Effort–Reward Imbalance model, ERI; e.g., Siegrist et al., 2009), which have been shown to relate to both work engagement and burnout (Kinnunen et al., 2008; Feldt et al., 2013).

In sum, we have set the following hypotheses based on previous research on nature exposure and outdoor activities in nature, as mentioned earlier:

H1: We expect to find distinctive profiles of nature exposure and outdoor activity in nature that are characterized by different frequencies of nature exposure and a heterogeneity of outdoor activities in nature.
H2: Employees with a profile characterized by less frequent nature exposure and less varied outdoor activities in nature will report low occupational well-being.
H3: Employees with a profile characterized by more frequent nature exposure and more varied outdoor activities in nature will report high occupational well-being.

## Materials and methods

### Data collection and participants

This research was conducted to investigate the relationship between visits to natural environments and occupational well-being among employees. The data were collected with an electronic survey, which included questions regarding employees' frequency and duration of visits to natural environments, their engagement in different types of outdoor activities in nature, occupational well-being, and demographic and work characteristics. The electronic link to the online survey was e-mailed to 3,260 employees of 13 public and private sector organizations between May and November 2016. The organizations were recruited directly, including the largest organizations in Central Finland and the Tampere region, with the help of two large occupational health services who forwarded the invitation for taking part in the study to the selected client organizations. The response rate was 24% (*N* = 783). Our study was carried out in accordance with the recommendations of the University of Jyväskylä's Ethics Committee and was given a research permit by the Tampere District Hospital. The registration number of the research permit is 430.

Of the participants, 78% were female, the average age was 47 years (*SD* = 10 years, range 21–70 years), and 65% of the participants had children. The participants' educational level was rather high as 56% held a university degree. Of the participants, 91% had full-time work and 76% worked regular daytime hours. Altogether, 17% of the participants were employed in a supervisory position. Of the participants, 35% were employed in a municipality, working in various public sector services. Other participants worked in various organizations, including social and health services (24%), education (21%), logistics and travel (12%), and design and engineering services (8%). The distribution of the participants in regard to their accessibility and exposure to nature is presented in Table 1.

**Table 1:** Percentages for variables describing participants' nature exposure and accessibility to nature areas.

**Nature exposure and accessibility**	**%**
**Frequency of nature visits during leisure time (1–7)**	**Summer/winter**
Never	0/1
Less than monthly	1/6
1–3 times per month	4/10
Once a week	7/14
2–3 times per week	18/26
4–6 times per week	26/18
Daily	44/25
**Duration of nature visits during leisure time (1–6)**	**Summer/winter**
Less than 15 min	1/3
15–30 min	6/15
30 min to 1 h	31/43
1–1.5 h	31/26
1.5–2 h	18/9
Over 2 h	13/4
**The distance to the nearest nature area from home (1–6)**	
Less than 100 m	64
100–300 m	24
300–500 m	6
500–1000 m	4
1–2 km	1
Over 2 km	1
**Frequency of visits to nature area at work (1–6)**	
No visit	67
Less than monthly	12
Monthly	4
Weekly	9
Almost daily	5
Daily	3
**Length of commute via nature (1–5)**	
None	37
Less than 500 m	21
500–1,000 m	16
1–1.5 km	9
Over 1.5 km	17

### Measures

#### Frequency of nature visits at work and leisure time

Frequency of nature visits at work and leisure time was measured with two separate questions. At the beginning of the survey, the participants were informed that our definition of green and nature environments includes areas such as parks, forests, fields, meadows, marshes, rocks, fells, beaches, waters, playgrounds, and playing fields. The first question related to nature visits during leisure time: “How often do you visit green and nature environments?” The participants indicated the frequencies of their visits separately for the summer season (May to September) and winter season (October to April) on a scale from *never* (1) to *daily* (7). Similarly, the second question related to the frequency of nature visits at work: “Do you spend time outside in green and nature environments at work?” The response scale ranged from *never* (1) to *daily* (6). The different response options are shown in Table 1.

#### Outdoor activities in nature

Outdoor activities in nature were enquired about with the question: “How do you normally use green and nature environments?” The participants selected the types of activities in nature they normally engage in from a given list of 16 activities (0 = *no*, 1 = *yes*) (Sievänen and Neuvonen, 2011). The list included a range of activities that described being in nature (e.g., enjoying scenery, relaxing, and dwelling), exercising in nature (e.g., walking and jogging, cycling, skiing), going on nature trips and travels (e.g., spending time at a summer cottage, boating), and the use of nature's resources (e.g., picking berries and mushrooms, hunting and fishing).

#### Work engagement

Work engagement was measured with the 9-item Utrecht Work Engagement Scale (Schaufeli et al., 2006; Seppälä et al., 2009). The dimensions of vigor (e.g., “At my work, I feel bursting with energy”), dedication (e.g., “I am enthusiastic about my job”) and absorption (e.g., “I feel happy when I am working intensely”) were all measured with three items. The rating scale ranged from *never* (1) to *daily* (7). The Cronbach's alphas were: vigor α = 0.89, dedication α = 0.91, and absorption α = 0.86. The three dimensions of work engagement were included separately in the analyses.

#### Burnout

Burnout was measured with the Bergen Burnout Inventory with nine items (BBI−9; Salmela-Aro et al., 2011) whose factorial invariance has been supported across organizations and measurement times (Feldt et al., 2014). The dimensions of exhaustion (e.g., “I am snowed under with work”), cynicism (e.g., “I feel dispirited at work and I think of leaving my job”), and inadequacy (e.g., “I frequently question the value of my work”) were all measured with three items. The rating scale ranged from *totally disagree* (1) to *totally agree* (6). The Cronbach's alphas were: exhaustion α = 0.69, cynicism α = 0.85, and inadequacy α = 0.80. The three dimensions of burnout were included separately in the analyses.

#### Covariates

The following demographic characteristics were included in the analyses: age (continuous), gender (0 = female, 1 = male), education (0 = no university degree, i.e., low education; 1 = university degree, i.e., high education), and having children (0 = no, 1 = yes). The following work factors were also included: being a supervisor (0 = no, 1 = yes), working a regular day shift (0 = no, 1 = yes), working hours per week (continuous), being in full-time work (0 = no, 1 = yes), and having a white-collar job (0 = no, 1 = yes).

Additional work-related factors of effort and reward were used as covariates. Participants evaluated their job stressors with the Effort-Reward Imbalance Scale (ERI scale; Siegrist et al., 2009). The original, longer version of the ERI scale has been validated in Finland (Rantanen et al., 2013). Our study's participants evaluated their efforts with three items (e.g., “I have constant time pressure due to a heavy workload”) and rewards with seven items (e.g., “I receive the respect I deserve from my superiors”). The response scale ranged from *totally disagree* (1) to *totally agree* (4). The Cronbach's alphas were: effort α = 0.69, and reward α = 0.79.

Further questions regarding nature exposure and accessibility were included in the survey: the duration of the nature visits during leisure time in summer and winter, the distance to the nearest natural area from home, and the length of the commute via nature. These variables did not contribute to the variability between participants and therefore were not included in the Latent Profile Analysis. Instead, these variables were taken into consideration as covariates in the Analyses of Covariance, since they related to well-being measures with the exception of the length of the nature visits during leisure time in the summer, which was not shown to be a significant covariate.

### Analyses

Pearson correlation coefficients were calculated to show the relationships between the nature-related variables and dimensions of work engagement and burnout. Latent Profile Analysis (LPA) was used to identify different subsamples of employees in regard to their nature exposure and outdoor activities in nature. The profiles were identified with the three questions specified earlier, that is, the frequency of nature visits during leisure time in summer and winter, the frequency of nature visits at work, and the types of outdoor activities in nature environments during leisure time. The analysis was performed using the Mplus statistical package (Version 7.3) with maximum likelihood estimation (MLE).

Deciding the number of profiles was based on several fit indices (Jung and Wickrama, 2008). First, the Bayesian Information Criterion (BIC), the Vuong-Lo-Mendell-Rubin (VLMR) test, the Lo-Mendell-Rubin test (LMR), and the Bootsrapped Likelihood Ratio Test (BLRT) were calculated. The lower the BIC values are, the better the model is. In the VLMR, LMR and BLRT, *p* < 0.05 indicates that *k* profiles are sufficient compared to *k* + 1 profiles. Second, a good solution was seen to be indicated when there was successful convergence, a high entropy value (range 0–1) and at least 1% of the participants in a profile. The third and most important criterion was that the identified profiles are meaningful.

We conducted the following analyses. First, the identified profiles were compared with *t*-tests (continuous variables) and χ^2^-tests (categorical variables) in regard to demographic, work- and nature-related factors. Second, separate Analyses of Covariance (ANCOVAs) were run for each well-being outcome in order to evaluate how the identified profiles are related to occupational well-being (i.e., vigor, dedication, absorption, exhaustion, cynicism, inadequacy). In these analyses, only the statistically significant covariates were included in the final models. In other words, first all the covariates related to demographic characteristics, work-related factors and nature-related factors (listed in the Measures section) were included in the models, and then, one by one, all of the statistically non-significant covariates were removed.

## Results

### Descriptive results

Table 2 depicts the intercorrelations between the nature-related factors and dimensions of work engagement and burnout. More frequent visits to nature environments during summer and winter, as well as shorter distances from home to nature environments, related to higher vigor, dedication and absorption. Also, longer visits to nature environments during the winter and longer commutes via nature related to higher vigor and absorption. More frequent visits to nature environments at work only related to dedication. Of the nature-related factors, only more frequent visits to nature environments at work related to lower burnout on the dimension of cynicism. The length of the visits to nature environments during summer was not related to any of the occupational well-being indicators.

**Table 2:** Pearson correlation coefficients for nature-related variables and indicators of occupational well-being.

**Variables**	**1**	**2**	**3**	**4**	**5**	**6**	**7**	**8**	**9**	**10**	**11**	**12**
**NATURE-RELATED FACTORS**												
1. Frequency of visits in winter (1 = Never−7 = Daily)^A^												
2. Frequency of visits in summer (1 = Never−7 = Daily)^A^	0.77^***^											
3. Duration of visits in winter (1 = Less than 15 min−6 = Over 2 h)^B^	0.16^***^	0.07^*^										
4. Duration of visits in summer (1 = Less than 15 min−6 = Over 2 h)	0.02	0.08^*^	0.62^***^									
5. Distance to nature area (1 = Less than 100 meters−6 = Over 2 km)^B^	−0.24^***^	−0.25^***^	−0.02	0.01								
6. Frequency of visits to nature area at work (1 = No visit−6 = Daily)^A^	0.19^***^	0.14^***^	0.05	0.05	−0.08^*^							
7. Length of commute via nature (1 = None−5 = Over 1.5 km)^B^	0.22^***^	−0.23^***^	0.12^**^	0.08^*^	−0.10^**^	0.10^*^						
**OCCUPATIONAL WELL-BEING**												
Vigor (1–7)	0.16^***^	0.13^***^	0.10^**^	0.04	−0.10^**^	0.06	0.09^*^					
Dedication (1–7)	0.13^***^	0.12^**^	0.06	0.01	−0.08^*^	0.09^*^	0.08	0.77^***^				
Absorption (1–7)	0.09^*^	0.09^*^	0.09^*^	0.04	−0.10^**^	0.05	0.08^*^	0.69^***^	0.72^***^			
Exhaustion (1–6)	−0.03	−0.03	−0.02	0.02	0.01	0.03	−0.04	−0.43^***^	−0.32^***^	−0.22^***^		
Cynicism (1–6)	−0.05	−0.05	−0.02	0.00	0.04	−0.08^*^	−0.05	−0.66^***^	−0.68^***^	−0.54^***^	0.55^***^	
Inadequacy (1–6)	−0.04	−0.04	0.00	0.03	0.05	−0.05	−0.05	−0.59^***^	−0.58^***^	−0.45^***^	0.53^***^	0.81^***^

* *p < 0.05*;

** *p < 0.01*;

*** *p < 0.001*.

A *Nature-related variable included in the LPA*.

B *Nature-related variable used as a covariate in the ANCOVAs*.

### Identifying profiles of nature exposure and outdoor activities

Table 3 presents the results of the LPA analyses for alternative multi-group solutions (1–5). The six-profile solution did not converge, despite the modifications to the number of random starts and starting values. Of the alternative profiles, the BIC, VLMR, and LMR supported a five-profile solution. Entropy was higher in the two-profile solution but acceptable in every solution. In the five-profile solution, the smallest profile included 8.4% of the participants. Thus, the solution with five profiles best fulfilled the statistical criteria and was selected.

**Table 3:** The results of latent profile analyses of nature exposure and outdoor activity.

**# of profiles**	**Log-likelihood**	**BIC**	**VLMR *p*-value**	**LMR *p*-value**	**BLRT *p*-value**	**Entropy**	**Proportions, *n* (%)**
1	−11194.19	22534.96	–	–	–	–	783 (100)
2	−10774.93	21829.72	0.000	0.000	0.000	0.830	213 (27.2) 570 (72.8)
3	−10482.60	21378.31	0.000	0.000	0.000	0.894	179 (22.9) 143 (18.3) 461 (58.9)
4	−10328.24	21202.86	0.000	0.000	0.000	0.828	172 (22.0) 144 (18.4) 161 (20.6) 306 (39.0)
5	−10197.31	21074.25	0.001	0.001	0.000	0.856	171 (21.8) 150 (19.2) 99 (12.6) 297 (37.9) 66 (8.4)
6	Did not converge						

**Figure 1:**
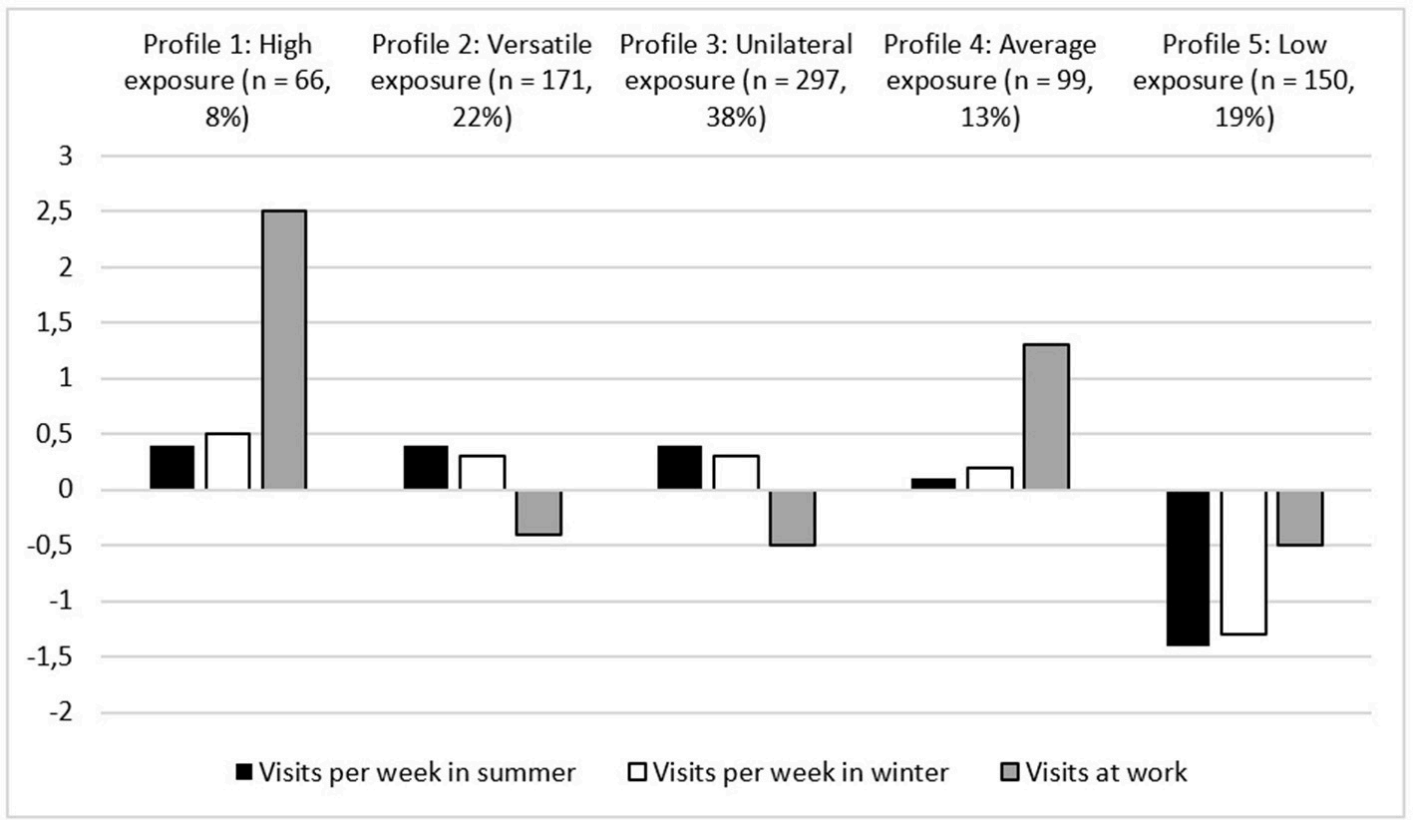
The five-profile solution of the LPA showing the frequency of nature of visits per week in summer and winter during leisure time and the frequency of visits at work.

In Figure 1, three of the variables in the LPA model are illustrated (i.e., frequency of nature visits during leisure time in summer and winter, and frequency of nature visits at work). These variables are standardized as they were measured with different scales. As can be seen in Figure 1, the frequency of the nature visits in summer and winter is the highest in Profile 1, the lowest in Profile 5, and at an average level in Profile 4. The frequency of nature visits at work is the highest in Profiles 1 and 4. In addition to the three profiles that show only differences in their levels of nature exposure, two other profiles (2–3) were identified. Table 4 shows that the profiles differed in terms of demographic, work- and nature-related variables. The participants in Profiles 1, 2, and 3 visited nature environments in summer significantly more often than did the participants in Profiles 4 and 5. Furthermore, the participants in Profile 5 visited nature environments in winter significantly less often than did all of the other participants. The participants in Profile 1 visited nature environments at work more often than did all of the other participants; and furthermore, participants in Profile 4 visited nature environments at work more often than did the participants in Profiles 2, 3, and 5.

**Table 4:** Comparison of the profiles: either the percentage or mean is presented with the related statistical testing.

	**Profiles**					**Statistical test**
	**High exposure**	**Versatile exposure**	**Unilateral exposure**	**Average exposure**	**Low exposure**	
**VARIABLES IN THE LPA (RANGE; SD)**						
Frequency of visits in summer (1–7; 1.22)	6.42	6.40	6.42	6.03	4.18	*p* < 0.001; 1, 2, 3 > 4, 5
Frequency of visits in winter (1–7; 1.53)	5.89	5.67	5.62	5.39	3.07	*p* < 0.001; 1, 2, 3, 4 > 5
Frequency of visits at work (1–6; 1.42)	5.39	1.20	1.13	3.70	1.13	*p* < 0.001; 1 > 4 > 2, 3, 5
**DEMOGRAPHICAL CHARACTERISTICS**						
**Gender**						*p* < 0.001
Female	83.3	78.9	80.8	85.9^A^	64.7	
Male	16.7	21.1	19.2	14.1	35.3^A^	
Education						*p* = 0.001
Low	69.7^A^	38.8	42.2	44.4	43.3	
High	30.3	61.2	57.8	55.6	56.7	
Children						*p* = 0.047
No	39.4	26.3	37.4	33.3	41.3	
Yes	60.6	73.7^A^	62.6	66.7	58.7	
Age in years	44.09	50.66	47.48	44.60	46.55	*p* < 0.001; 2 > 1, 3, 4, 5
**WORK-RELATED FACTORS**						
Supervisor						*p* = 0.016
No	89.4	78.9	81.1	92.9^A^	80.8	
Yes	10.6	21.1	18.9	7.1	20.0	
Regular day shift						*p* < 0.001
No	42.4^A^	13.5	18.5	52.5^A^	19.3	
Yes	57.6	86.5^A^	81.5^A^	47.5	80.7	
Full-time work						*p* = 0.333
No	7.6	10.5	10.4	4.0	8.0	
Yes	92.4	89.5	89.6	96.0	92.0	
White-collar worker						*p* < 0.001
No	83.3^A^	47.6	60.9	77.8^A^	54.7	
Yes	16.7	52.4^A^	39.1	22.2	45.3	
Working hours/week	37.84	39.69	39.01	38.89	39.77	*p* = 0.403
Effort	2.86	2.98	2.86	2.85	2.86	*p* = 0.224
Reward	2.32	2.45	2.43	2.48	2.43	*p* = 0.393
**NATURE-RELATED FACTORS**						
Duration of visits in winter (1–6; 1.06)	3.44	3.60	3.27	3.33	3.04	*p* < 0.001; 2 > 3, 5
Distance to natural area (1–6;.92)	1.32	1.40	1.53	1.48	1.95	*p* < 0.001; 5 > 1, 2, 3, 4
Length of commute via nature (1–5; 1.48)	2.62	2.87	2.41	2.78	1.96	*p* < 0.001; 5 < 1, 2, 4; 2 > 3

A *This class is over-represented in this profile*.

The participants in Profiles 2 and 3 are similar in regard to these three variables: they visited nature environments during leisure time more often than did the average of the sample, but the frequency of their nature visits at work was less than was the case for the average of the sample. The reason why the LPA identified Profiles 2 and 3 as separate is due to the fact that the participants in these profiles differ in their patterns of activities in nature environments (see Table 5). In Profile 2, the participants were active in nature environments in various ways: they spent time in nature in a number of different ways, such as enjoying the scenery, relaxing, gardening, sunbathing, and swimming. They exercised in nature by walking and jogging, cycling, and skiing. They also spent time in their cottage, went boating, and picked berries and mushrooms. In contrast, in Profile 3, the most common activities were enjoying the scenery and nature, and walking and jogging. Moreover, the participants in Profiles 1, 4, and 5 were also rather narrow in their activities, since they mainly enjoyed the scenery and nature, relaxed, walked, and jogged.

**Table 5:** Percentages per profile of participants engaging in each of the different outdoor activities in nature environments during leisure time (activities in which over 50% of participants in each profile engaged are marked in bold).

**Outdoor activity**	**High exposure**	**Versatile exposure**	**Unilateral exposure**	**Average exposure**	**Low exposure**
**BEING IN NATURE**					
Enjoy scenery and nature	**88**	**93**	**82**	**95**	**79**
Relaxing and dwelling	**73**	**84**	50	**71**	**54**
Sunbathing and swimming	39	**71**	28	48	43
Gardening	41	**70**	38	43	25
Photographing, painting or observing nature	33	33	22	28	16
**EXERCISE IN NATURE**					
Walking and jogging	**88**	**100**	**87**	**90**	**75**
Cycling	49	**77**	41	38	35
Skiing	33	**71**	28	34	24
Walking and playing with children	46	47	23	34	18
Walking with my pet	41	30	43	37	7
Playing	15	18	5	11	8
**NATURE TRIPS AND TRAVELS**					
Spending time at cottage	35	**63**	24	35	35
Boating	27	**58**	8	27	15
Camping	17	37	8	24	7
**THE USE OF RESOURCES IN NATURE**					
Picking berries and mushrooms	**59**	**92**	50	49	39
Hunting and fishing	14	28	6	16	15

Based on these results, the profiles can be described as follows: Profile 1 = *High exposure* (*n* = 66; 8%), describing frequent nature visits at work and during leisure time; Profile 2 = *Versatile exposure* (*n* = 171; 22%), describing frequent nature visits and versatile activity during leisure time combined with less frequent nature visits at work; Profile 3 = *Unilateral exposure* (*n* = 297; 38%), describing frequent nature visits but unilateral activity during leisure time combined with less frequent visits at work; Profile 4 = *Average exposure* (*n* = 99; 13%), describing average frequency of nature visits at work and during leisure time; Profile 5 = *Low Exposure* (*n* = 150; 19%), describing less frequent nature visits at work and during leisure time.

### Profiles of nature exposure and activity in relation to occupational well-being

Tables 6, 7 present the results related to the ANCOVAs: specifically, the estimated marginal means for the profiles in regard to occupational well-being factors (Table 6) and the final ANCOVA models with only statistically significant covariates (Table 7). The profiles differed in vigor, dedication, and cynicism after the statistically significant covariates were taken into account, explaining 1–3% of the variance in the well-being variables. In addition, the differences between the profiles were marginally significant in relation to professional inadequacy.

**Table 6:** Estimated marginal means (and standard errors) of well-being outcomes for the profiles (see Table 7 for covariates used).

	**Profiles**					***F*-test**
	**High exposure**	**Versatile exposure**	**Unilateral exposure**	**Average exposure**	**Low exposure**	
Vigor	6.01 (0.13)	5.89 (0.08)	5.71 (0.06)	5.57 (0.11)	5.37 (0.09)	*F*_(4, 743)_ = 6.59, *p* < 0.001
Dedication	6.34 (0.13)	6.06 (0.08)	5.93 (0.06)	5.97 (0.11)	5.62 (0.09)	*F*_(4, 775)_ = 6.08, *p* < 0.001
Absorption	6.04 (0.14)	5.81 (0.09)	5.69 (0.06)	5.67 (0.12)	5.61 (0.09)	*F*_(4, 746)_ = 1.99, *p* = 0.093
Exhaustion	2.69 (0.10)	2.81 (0.06)	2.80 (0.05)	2.82 (0.08)	2.82 (0.07)	*F*_(4, 767)_ = 0.31, *p* = 0.870
Cynicism	1.87 (0.11)	2.19 (0.07)	2.18 (0.05)	2.15 (0.09)	2.33 (0.07)	*F*_(4, 775)_ = 3.16, *p* = 0.014
Inadequacy	2.17 (0.12)	2.41 (0.08)	2.44 (0.06)	2.40 (0.10)	2.57 (0.08)	*F*_(4, 775)_ = 1.96, *p* = 0.099

**Table 7:** The final results of six separate analyses of covariance with significant covariates: parameter estimates (Unstandardized B) are reported in order to show the direction of the relationship.

	**Vigor**	**Dedication**	**Absorption**	**Exhaustion**	**Cynicism**	**Inadequacy**
**DEMOGRAPHICAL COVARIATES**						
Age in years	–	–	–	–	–	–
Male	−0.34^***^	−0.29^**^	−0.29^**^	–	–	–
High education	−0.15^*^	–	–	–	–	–
Having children	–	0.21^**^	–	–	−0.13^*^	−0.18^*^
**WORK-RELATED COVARIATES**						
Supervisor position	0.30^***^	–	–	–	–	–
Regular day shift	–	–	0.25^**^	–	–	–
Full-time work	–	–	–	–	–	–
White-collar worker	–	–	–	–	–	–
Working hours	–	–	–	0.02^***^	–	–
Effort	−0.14^*^	–	0.17^*^	0.81^***^	0.24^***^	0.23^***^
Reward	0.98^***^	1.06^***^	0.95^***^	−0.52^***^	−1.03^***^	−1.34^***^
**NATURE-RELATED COVARIATES**						
Duration of visits in winter during leisure time	0.13^***^	–	0.09^*^	–	–	–
Distance to natural area	−0.11^*^	–	−0.15^**^	–	–	–
Length of commute via nature	–	–	–	–	–	–
**PROFILES**						
High exposure	0.63^***^	0.71^***^	0.43^*^	−0.12*^ns^*	−0.47^***^	−0.40^**^
Versatile exposure	0.52^***^	0.44^***^	0.19*^ns^*	−0.01*^ns^*	−0.15*^ns^*	−0.15*^ns^*
Unilateral exposure	0.33^**^	0.31^**^	0.08*^ns^*	-0.02*^ns^*	−0.15*^ns^*	−0.13*^ns^*
Average exposure	0.19*^ns^*	0.35^*^	0.06*^ns^*	0.00*^ns^*	−0.18*^ns^*	−0.17*^ns^*
Low exposure (reference)	–	–	–	–	–	–
Adjusted *R*^2^	0.29	0.24	0.20	0.38	0.31	0.38
η^2^ (profiles)	0.03^***^	0.03^***^	0.01	0.00	0.02^*^	0.01

*Profile 5 was selected as the reference category*.

ns *p > 0.10*;

* *p < 0.05*;

** *p < 0.01*;

*** *p < 0.001*.

*Non-significant covariates were removed from the final model*.

In order to reduce the number of pairwise comparisons, only the low exposure profile was compared to the other profiles (instead of comparing all profiles to each other). The participants in the high exposure profile reported higher vigor (β = 0.63, *p* < 0.001), dedication (β = 0.71, *p* < 0.001) and absorption (β = 0.43, *p* < 0.05), and lower cynicism (β = − 0.47, *p* < 0.001) and inadequacy (β = − 0.40, *p* < 0.01) than did the participants in the low exposure profile. Moreover, the level of vigor was lower in the low exposure profile compared to that of the participants in the versatile nature exposure profile (β = 0.52, *p* < 0.001) and unilateral exposure profile (β = 0.33, *p* < 0.001). The participants in the low exposure profile also reported lower levels of dedication compared to the participants in the versatile exposure profile (β = 0.44, *p* < 0.001), the unilateral exposure profile (β = 0.31, *p* < 0.01) and the average exposure profile (β = 0.35, *p* < 0.05). In the Bonferroni corrections, the *p*-value is multiplied by the number of pairwise comparisons. In this case, we have four pairwise comparisons. The *p*-values under 0.01 remain under 0.05 and *p*-values under 0.05 remain under 0.1 when Bonferroni corrections are calculated. The only occurrence of *p* < 0.05 was for the difference between the participants in the low exposure and high exposure profile regarding absorption, as well as for the difference between the participants in the low exposure profile and average exposure profile regarding dedication. These *p*-values may become marginally significant when Bonferroni corrections are taken into account.

Additionally, the versatile and unilateral exposure profiles were compared to see whether the diversity of the outdoor activities plays a role in well-being. However, these two profiles did not differ in regard to any of the well-being variables (results not reported here).

## Discussion

Our present research findings offer a valuable step forward from previous studies by having utilized a person-centered approach to identify profiles of nature exposure and outdoor activity in nature among a range of Finnish employees. As expected in our hypotheses, there was heterogeneity in the levels of nature exposure and outdoor activities in nature, which was captured by five profiles of nature exposure and outdoor activity in nature. Our hypothesis regarding the relationship of the profiles of nature exposure and outdoor activity in nature with occupational well-being received support, since the profiles were associated with burnout and work engagement.

### Favorable profiles of nature exposure and outdoor activity in nature in relation to occupational well-being

The participants in the high, versatile and unilateral exposure profiles reported on average 4–6 weekly visits to nature environments in the summer months and 2–3 weekly visits in the winter months during leisure time. These participants visited nature environments in the summer more frequently than did the participants in the average and low exposure profiles. Furthermore, these participants differed in the frequency of their nature visits from the overall one-fifth of the participants who belonged to the profile of low exposure, who visited nature environments during leisure time once a week in the summer and 1–3 times during the month in the winter, on average. It is therefore an encouraging finding that about 68% of participants belonged to the profiles of high, versatile, and unilateral nature exposure profiles, which can be considered as favorable profiles regarding nature exposure and outdoor activity in nature. These profiles can also be considered as favorable profiles in terms of occupational well-being, since these participants reported higher work engagement in the dimensions of vigor and dedication than did the participants in the profile of low exposure.

Participants in the versatile exposure profile visited natural environments during leisure time and at work as frequently as did the participants in the unilateral exposure profile, but there were differences in the range of their activities in nature environments. Participants who had versatile activities spent time on being in nature (e.g., enjoying scenery and nature, relaxing and dwelling), exercising in nature, engaging in nature trips and travels, and utilized resources of nature such as by picking berries and mushrooms. The participants with unilateral activity typically engaged in less varied activities: enjoying and being in nature as well as walking. However, participants in both profiles were similar from the perspective of occupational well-being.

The association between the profiles and occupational well-being was highlighted in relation to the vigor and dedication dimensions of work engagement. The participants in the profiles of favorable nature exposure and outdoor activity in nature reported higher vigor and dedication compared to the participants in the low exposure profile. These findings are in line with various research showing the well-being effects of natural environments (e.g., van den Berg et al., 2015). The findings also parallel recent longitudinal research on employees, in which physical activity in nature during leisure time was found to contribute to their vitality (Korpela et al., 2017). Our research provides further evidence that more frequent visits to natural areas can be linked with positive motivational work-related states. On the basis of the ART (Kaplan, 1995) and SRT (Ulrich et al., 1991), nature environments may improve concentration and promote positive affect that presumably play a part in employees' resources for experiencing vigor and dedication at work.

The relationships between the profiles and burnout was less prevalent. The participants belonging to the high exposure profile reported lower cynicism and inadequacy than did the participants in the low exposure profile. In fact, the participants in the high exposure profile reported on average the lowest burnout in all dimensions of burnout and in conjunction with the highest work engagement.

The participants in the high exposure profile are of particular interest, since they differed in the frequency of their nature visits at work from the other participants in the favorable profiles of nature exposure and outdoor activity in nature (the profiles of versatile and unilateral exposure). The participants in the high exposure profile reported being exposed to nature environments on average almost daily during their workday. These results suggest that more frequent exposure to natural environments at work can have beneficial associations with occupational well-being. However, it should be noted that a high frequency of professional nature visits does not necessarily lead to well-being benefits, since participants in the average exposure profile reported only higher dedication in comparison to the participants in the low exposure profile (see below).

The participants in the high exposure profile were more likely to have a lower education, a blue-collar position, and to work irregular day shifts. The other participants in the favorable nature exposure profiles (the profiles of versatile and unilateral exposure) were more likely to work regular day shifts. In addition, the participants in the versatile exposure profile were more likely to be white-collar workers. Based on these results, the participants who work in typical office jobs could gain a further boost to their occupational well-being by having access to more exposure to nature during their workday. This suggestion is supported by previous studies, which have shown that spending more time outdoors during work has beneficial associations with employee well-being (e.g., Gilchrist et al., 2015), such as through the effects of increased vitality and decreased fatigue as well as blood pressure after a park walk during the lunch break (de Bloom et al., 2017; Torrente et al., 2017).

### The profiles of average and low nature exposure in relation to occupational well-being

The results of our study also revealed a profile representing an average level of nature exposure. These participants visited nature environments during leisure time on average less often in the summer than did the participants in the favorable profiles of exposure and outdoor activity in nature, but more often than did the participants in the low exposure profile during the winter. The participants in this profile of average exposure were similar to the participants in the profile of high exposure in terms of the demographic characteristics. They were more likely to be women and working in irregular day shifts in blue-collar, non-supervisory positions. Also, their work entailed nature exposure more frequently than was the case with the participants in the versatile, unilateral, and low exposure profiles.

Overall, the participants in the average exposure profile reported an average level of occupational well-being as well as higher dedication than did the participants in the low exposure profile. On the basis of our findings, it is possible that the participants in the average exposure profile would benefit from more regular nature exposure during leisure time in order to promote higher-than-average occupational well-being. However, their shift work may restrict their possibility to do that.

It is worth noting that the participants in the average exposure profile reported visiting nature areas during work (similar to the participants in the high exposure profile). On the basis of previous empirical findings on forestry professionals (Von Lindern et al., 2013) and restoration theories (e.g., Kaplan and Kaplan, 1989), it could be that gaining a sense of being away may be difficult when the nature visits are work-related. The work-related nature experience may be different in quality and not as restorative as experiences during leisure time. To understand the differing well-being effects and somewhat contradictory findings concerning work-related nature experiences, more research on the relationship between professional nature exposure and well-being benefits is clearly needed. In particular, the elements of fascination and being away in restoration require further research in relation to the frequency of professional nature exposure. Different frequencies may, for example, offset or amplify experiences of being away and fascination in different ways, and could thus lead to added or diminished well-being benefits.

Participants in the low exposure profile, in turn, were more likely to be male, and they reported the lowest work engagement and highest burnout, on average. Our findings also show that these participants had the longest distance to travel to get from home to natural environments. The longer distance from home may restrict the accessibility to nature environments, which is a consideration that is in line with previous research indicating that a longer distance from home to nature environments reduces the number of nature visits (Neuvonen et al., 2007). From this perspective, it is recommendable to increase especially such individuals' exposure to nature. The proximity of nature environments and their accessibility depends not only on an individual's decisions but also on regional and environmental supply and planning. Ideally, nature environments should be located near enough to residential and work environments to act as a resource for health and general as well as occupational well-being.

It is interesting that the participants of all the profiles reported enjoying nature and natural scenery, being and relaxing in nature. Exercise included mainly walking and jogging. Furthermore, the participants in the low exposure profile reported outdoor activities in nature such as enjoying the scenery and nature, as well as walking and jogging. However, engaging in these outdoor activities reasonably regularly seems to be needed to achieve higher levels of well-being, especially vigor and dedication. This finding is in line with a previous study (e.g., de Vries et al., 2003), in which increasing physical activity was not the only explanation for health benefits of nature environments. The well-being effects of a nature environment itself can be significant to some extent, but increased physical activity increases the odds for better well-being.

### Study limitations and conclusions

Our study is subject to several limitations that should be acknowledged before making inferences based on these findings. First, owing to the relatively low response rate, the representativeness of the sample needs to be considered. It is possible that the participants who responded to the survey were more inclined to nature visits. Those participants who failed to respond, in turn, may be utilizing natural environments to a lesser extent. Therefore, the profile of low exposure may have incorporated a larger proportion of employees if the response rate had been higher.

Second, the relationships between nature exposure and occupational well-being should be investigated with a longitudinal, gender-balanced sample of employees in order to get a more representative picture of different development paths. It is possible that those employees who have better occupational well-being also have more resources enabling them to engage in outdoor activities more frequently. Therefore, on the basis of this cross-sectional study, inferences regarding causal relations of nature exposure and outdoor activity with occupational well-being cannot be made. Third, the study is based on questionnaire data, and thus additional objective data (e.g., register-based sickness absence) should be collected in order to avoid the limitations of self-report data and same-source bias. In terms of future directions, it would also be valuable to investigate how nature experiences differ in association with the workplace vs. leisure time, since nature exposure in association with work appeared to play a role in the profiles of nature exposure in this study.

In conclusion, these findings highlight how employees' levels of nature exposure and outdoor activities in nature can contribute to their work engagement and burnout. Frequent opportunities for nature exposure at work as well as during leisure time can be related to higher vigor and dedication, and in turn lower cynicism and professional inadequacy. In line with the theories on the restorative effects of nature environments (e.g., Ulrich et al., 1991; Kaplan, 1995), employees may seek to engage in various activities in nature to regain their cognitive and psychological resources. However, the current results extend far beyond the restorative environment theories by showing that the relation of nature exposure to occupational well-being exists on a more general experiential level than the short-term effects of stress and attention restoration described by the ART and SRT. It is conceivable that, for example, changes in vigor and dedication require not only recovery of cognitive and emotional resources, but also active emotion- and self-regulation (Korpela et al., 2015).

## Author contributions

KH has designed the research work, collected the data and contributed to the analyses and interpretation of the findings. She has drafted and revised the work and approved the submitted version. She is accountable for all aspects of the work. KT has designed the research work, performed the analyses and written the results section of the paper. She has drafted and revised the work and approved the submitted version. KS has designed the research work, collected data and contributed to writing the introduction and discussion sections of the paper. She has drafted and revised the work as well as approved the submitted version of the paper. KK has designed the research work, contributed to data collection, analyses and interpretation of the findings. He has drafted and revised the work and approved the submitted version. TF has designed the research work, contributed to data collection, analyses and interpretation of the findings. She has also drafted and revised the work and approved the submitted version. UK has designed the research work, contributed to data collection, analyses and interpretation of the findings. She has drafted, revised and approved the work.

### Conflict of interest statement

The authors declare that the research was conducted in the absence of any commercial or financial relationships that could be construed as a potential conflict of interest.
